# Prevalence and mitigation strategies of HIV/AIDS infection risks in Namibian tertiary education institutional hostels

**DOI:** 10.1080/17290376.2014.918521

**Published:** 2014-05-12

**Authors:** Roderick F. Zimba, Gilbert N. Likando

**Affiliations:** ^a^ PhD, is affiliated to the Department of Educational Psychology and Inclusive Education at the University of Namibia, Windhoek, Namibia; ^b^ PhD, is affiliated to the Department of Educational Foundations and Management at the University of Namibia, Windhoek, Namibia

**Keywords:** HIV/AIDS, Namibia, infection risks, the youth, tertiary institutional hostels, mitigation strategies, VIH/SIDA, Namibie, risques d'infection, jeunes gens, foyers résidentiels d'institutions de l'enseignement supérieur, stratégies de réduction

## Abstract

The purpose of this study was to investigate risk factors that could promote HIV infection amongst adolescents and young adults living in tertiary educational institutional hostels in Namibia. Employing structured questionnaires and focus group discussions, we sought to answer questions pertaining to factors, beliefs systems, values, traditions and sexual relations that could promote HIV infection in the student hostels. The data on these issues were gathered from 306 male and 314 female students aged 18–35 years living in eight hostels. Amongst other results, the data revealed that sexual promiscuity in the hostels was treated as the norm in the majority of cases, unauthorized access to hostel rooms by non-hostel dwellers was rampant, sexual harassment of female students by men who were under the influence of alcohol was reported to be common and there was general lack of support for victims of sexual abuse in the hostels. In addition, there was a general sense of insecurity in the hostels where more than 50% of the participants were afraid of being sexually attacked, some female hostel residents engaged in sexual activities for monetary and material gain and there was a general practice of older men from the community having sexual relations with young female hostel dwellers. To mitigate these and other risks it is recommended that there be provision of more HIV/AIDS prevention services, enhanced security, non-toxic entertainment (e.g. participation in sport and social clubs) and the banning of the sale of alcohol in student residences and on tertiary institution campuses. These and other results are discussed in the article and ways of mitigating the risks are proposed.

## Résumé

Le but de la présente enquête était d'examiner les facteurs de risques qui pourraient contribuer à l'infection au VIH parmi les adolescents et jeunes adultes qui sont logés dans les foyers des institutions de l'enseignement supérieur en Namibie. Avec l'aide de questionnaires structurés et de discussions de groupes ciblés, nous avons essayé de répondre à des questions concernant les facteurs, systèmes de croyances, valeurs, traditions er relations sexuelles qui pourraient contribuer à l'infection au VIH dans les foyers résidentiels des étudiants. Les données sur ces questions ont été recueillies à partir de 306 étudiants et 314 étudiantes âgés de 18 à 35 ans. Entre autres résultats, les données ont révélé que, dans la plupart des cas, la promiscuité sexuelle dans les foyers était considérée comme la norme, l'accès non autorisée aux chambres des foyers par des non-résidents était endémique, le harcèlement sexuel des étudiantes par des hommes sous l'influence de l'alcool était, il semble, un fait courant, il n'y avait généralement pas d'appui aux victimes d'abus sexuels dans ces foyers, il y avait un sentiment généralisé d'insécurité dans les foyers, et plus de la moitié des participants avaient peur d’être la cible des attaques sexuelles, que certaines étudiantes dans ces foyers se livraient à des activités sexuelles pour des raisons pécuniaires et matérielles, et qu'il y avait d'ordinaire une pratique où des hommes plus âgés dans la communauté entretenaient des relations sexuelles avec des étudiantes internes. Pour réduire tous ces risques aussi bien que d'autres, on recommande la mise à disposition de plus de services de prévention du VIH/SIDA, beaucoup plus de sécurité, divertissements sans boissons alcooliques et l'interdiction de la vente d'alcool dans les résidences des étudiants et sur les campus des institutions de l'enseignement supérieur. Ces résultats, ainsi que d'autres, sont examinés dans l'article, et des moyens de réduction des risques ont été proposés.

## Background

It is common knowledge that the risk of HIV infection varies according to the behaviour of individuals, gender, social-economic status and the risks inherent in living environments (Matlin & Spence 2000; Ojedokun & Balogun 2008). According to the 2nd Millennium Development Report of 2008, Namibia's major challenge in combating HIV has been described as ‘ … the need for change in sexual behaviors’ (National Planning Commission 2008:36). In response to this challenge and consistent with the country's Vision 2030 goals, a cross-sectoral and multi-level approach has been adopted by the Namibian Government in the fight against HIV/AIDS (MoHSS 2008).

It is also commonly held that education is an important deterrent to the spread of HIV infection. It is assumed that the more people are aware of how HIV and AIDS spread, the more they will be able to guard against the disease. For more than three decades, these assumptions have guided HIV/AIDS prevention programmes in many countries. We do not need deep insight to arrive at the conclusion that the majority of such programmes have yielded little impact in the way of halting or even slowing down the disease's devastating onslaught on many communities, particularly those in Sub-Saharan Africa. Instead of diminishing, HIV infection has been increasing on an annual basis, almost unabated, in many communities around the world. Consistent with this, Boler and Archer (2008) reported some statistics which projected that by the year 2015 there would be 60 million more new HIV infections worldwide than there were in 2007. Although the rate of new HIV infections globally has reduced, the UNAIDS Global Report for 2013 shows that in 2012 there were 1.9 million new infections amongst adults from low- and middle-income countries (UNAIDS 2013). Because we have not yet reached the zero-HIV infection stage, the 2008 prediction of Boler and Archer still holds. This is consistent with the statement that ‘Everyday more than 7000 individuals become newly infected with HIV – more than twice as many as the number of people who start on antiretroviral therapy each day’ (The Aids 2031 Consortium 2010).

Voices have been heard pointing to the danger of ‘mis-education’ and ‘mis-schooling’ in the fight against HIV infection (Kelly 2000; Mwape & Kathuria 2000; Zimba & Nuujoma-Kalomo 2002). This danger is that educated people and educational settings can in fact promote HIV infection which is contrary to received wisdom (Stremlau & Nkosi 2001). Mwape and Kathuria (2000) reported that despite a high degree of awareness about HIV and AIDS, University of Zambia students engaged in risky sexual behaviours. These included having multiple partners, engaging in promiscuous activities, usually under the influence of alcohol and meeting financial obligations by offering sexual favours without exercising any form of discretion. Kelly (2000) described how Zambian learners in boarding schools could be exposed to HIV infection. Similarly, Ojedokun and Balogun (2008) reported that in developing countries such as Nigeria, premarital sexual intercourse was common among adolescents and college students. Consistent with all this, Zimba and Nuujoma-Kalomo (2002) reported findings on how Namibian learners residing in school hostels were at risk of HIV infection. These findings are also consistent with recent reports that show that HIV/AIDS prevalence in Namibia remains high despite all the interventions (National Planning Commission 2008). In 2007 alone, it was estimated that the total number of new infections was 15,700 and 43% of these new infections occurred amongst young people between the ages of 15 and 24 (National Planning Commission 2008:36). This persistence in the increase of HIV infections among young people seems to suggest that although they have heard about the HIV/AIDS epidemic, this awareness is not translated into strategies of how to protect themselves (Bezuidenhout & Summers 2007).

Apparent explanations of this state of affairs have been provided by some researchers. For instance, in examining the HIV/AIDS situation amongst adult learners in Botswana, Preece and Ntseane (2003) argued that there was an urgent need to deal with adverse attitudes, beliefs, behaviours and power imbalances between genders that could promote the spread of HIV/AIDS. Consistent with this argument, Appiah-Donyina's (2002) research in West Africa acknowledged the influence of cultural practices and gender-related factors in the spread of HIV infections.

Notwithstanding the above observations, we are aware that the education sector has an important role to play in combating HIV and AIDS. These include influencing changes in social attitudes and cultural norms acquired by young people in the family, from peers, religion and the media (Matlin & Spence 2000) as well as in imparting relevant skills and values to enable the youth to tackle the challenges posed by the pandemic (Boler & Archer 2008; Kirby, Laris & Rolleri 2005). What remains unclear is the actual nature of risks, attitudes and cultural norms that young people display in practice when interacting with one another in living environments such as University hostels.

Although Clerke (2008) argued that issues of risky sexual relationships and sexual favours were not new in educational institutions, his support of legal frameworks to guide practice and mitigate problems emanating from HIV/AIDS risks should be based on research evidence. The search for such evidence was one of the main reasons for undertaking the present study.

## Research problem

Although several studies have been conducted in the Namibian education system on the spread of HIV and AIDS in general and its impact on the workforce in particular (Castro, Duthilleul & Caillods 2007), no in-depth study has been carried out to understand factors that might promote HIV infection in settings such as hostels at tertiary educational institutions. We think that in the fight against HIV infection, it is important to understand these factors. According to Zimba and Mostert (1993) and Zimba and Nuujoma-Kalomo (2002), these factors can be in the form of hostel overcrowding, lack of safety in ablution facilities, the manner in which basic needs for food, shelter and clothing are met by hostel residents, general safety and security in the hostels, risk of sexual abuse in the hostels and lack of guidance and psychosocial support. In this study, we sought to find out whether HIV infection risks that pertained to such factors could be expressed by students residing in hostels at the University of Namibia (UNAM), the Polytechnic of Namibia and at the then Colleges of Education. Moreover, we wished to find out beliefs, values, traditions and sexual relational practices amongst students that might underlie the risk factors. Furthermore, we wished to learn from the respondents about what the tertiary institutions could do to mitigate the impact of these factors.

## Research questions

We addressed the following research questions in the study:

1. What factors may promote HIV infection amongst students residing in tertiary education institutional hostels in Namibia?
2. What beliefs, values, traditions and the practised sexual relations amongst students may underlie the risky factors?
3. What should these tertiary institutions do to mitigate the impact of these factors?

## Significance of the study

Although a number of studies on HIV/AIDS awareness, HIV infection risks, attitudes and practices that may promote HIV infection have been conducted at tertiary institutions in Namibia, we are unaware of systematic and in-depth studies on factors that promote HIV infection in the hostels of these institutions. We are not well informed about beliefs, cultural values and practices that facilitate HIV infection in student hostels at the UNAM, the Polytechnic of Namibia and at the former Colleges of Education. This investigation makes available findings on these and other issues. Furthermore, these findings form an important basis for strategies to mitigate the impact of HIV and AIDS risk factors prevalent in tertiary education institutions' hostels.

## Methodology

#### Research design

Both quantitative and qualitative methods were used in this study. As a quantitative method, questionnaires were used to explore various matters pertaining to students' thought patterns, behaviours, attitudes, beliefs, values, sexual practices and hostel living conditions that could promote HIV infection and the spread of AIDS, whilst as a qualitative method, focus group discussions were used to capture students' views and experiences on factors that could promote HIV infection in the hostels.

### Population

The population of this study consisted of all hostel residents from the UNAM, the Polytechnic of Namibia and from the then four Colleges of Education in Namibia that merged with the UNAM in 2011.

#### Sample

A sample of 629 hostel residents was systematically drawn from the population. To avoid sampling bias, we proportionally sampled participants according to the number of hostel residents in an institutional hostel. The more hostel residents there were in an institution, the more they were represented in the sample and vice versa (see Table 1). In addition, all tertiary institutions in the targeted population were included in the sample, and male and female participants were represented in the sample in almost equal proportion. These participants were drawn from all sectors of the socio-economic spectrum and from all Namibian ethnic groups. Whereas 49% of the participants were male, 51% were female. About 83% of the participants were between the ages of 18 and 25 years. Whereas 98.6% of the sample consisted of Namibian students, 1.4% of the sample was made up of non-Namibian students. Because participation in the study was on a voluntary basis, we thought that the sample size was adequate.

**Table 1.** Questionnaire administration.

Institution	No. of questionnaires
Caprivi College of Education Hostel	72
Rundu College of Education Hostel	95
Ongwediva College of Education Hostel	175
Windhoek College of Education Hostel	104
Polytechnic of Namibia Hostel	61
University of Namibia Hostel (main Campus, Neudamm and Ogongo campuses)	122
Total	629

#### Research instruments

To collect data, researchers used a structured questionnaire, focus group interview questions and a tape recorder. The questionnaire and the interview schedule were original instruments that were constructed by the researchers.

#### Pre-testing research instruments

To enhance their reliability and validity, the questionnaire items and interview questions were pre-tested on some UNAM hostel residents who did not participate in the main study. This was meant to reduce errors caused by vagueness and errors in the instruments.

#### Results of the pre-test

The results of the Pre-test demonstrated that all the questions were understood by the respondents. On the basis of these results no items in the research instruments were revised.

#### Procedure

After obtaining official access to the hostels, questionnaires were administered to 629 hostel residents at the identified tertiary educational institutions. All those who received questionnaires filled them in and returned them. None of the respondents who were asked to participate in the study declined to respond to the questionnaire. This was followed by focus group discussions that were conducted amongst 191 hostel residents. Based on the proportion of students in the target population, the questionnaires were administered as provided in Table 1.

We conducted focus group interviews as given in Table 2.

**Table 2.** Focus group interviews.

Institution	Residents interviewed
Caprivi College of Education Hostel	25
Rundu College of Education Hostel	39
Ongwediva College of Education Hostel	63
Windhoek College of Education Hostel	7
Polytechnic of Namibia Hostel	7
University of Namibia Hostel	50
Total	191

The focus group discussion participants were randomly selected from the 629 participants who responded to the questionnaire. At each institution, focus groups of six to eight members were formed. Overall, there were 24 focus groups. Each group consisted of male and female participants. Each session of the focus group discussion took approximately 30–45 minutes. During the discussion, one researcher asked questions and guided the discussions while the other researcher recorded the responses on tape and in writing. With the consent of participants, deliberations of the focus groups were tape-recorded at all the sampled institutions. Observations of physical hostel settings, participants and interactions amongst them were informally undertaken.

#### Ethical considerations

Consistent with Jarvis’ (1997) research ethics guidelines, researchers in this study took into consideration the respondent's right to anonymity in the process of collecting data. They made sure that informed consent was obtained before responses were tape recorded. After obtaining official access to the hostels from the management of all institutions involved, the purpose of the study was explained to all the respondents before the structured questionnaires were administered and focus group interviews were conducted.

At the beginning of each data collection session, researchers explained the process of responding to the questionnaire and interview questions. They also informed participants that their responses were confidential and that they could withdraw from the exercise at any time they felt they needed to do so. Because the project on which this article is based was funded by the UNAM, it was approved by the institution's Research and Publications Committee. In addition, we obtained permission to conduct the study at the various campuses from the University's Pro Vice Chancellor: Academic Affairs and Research as the University did not have a Research Ethics Committee at the time. It now does have such a committee.

#### Data analysis

Whereas the questionnaire data were analysed using descriptive statistics as we did not seek to make comparisons due to demographic variables, interview data from focus group discussions were transcribed, categorized and content analysed. Logical reflections were used to discern messages on HIV risks from observations that were undertaken. These included descriptions of key hostel events, various hostel settings, hostel processes and key dynamics of hostel life. In other words, some meanings were discerned from the data by relating, for example, the nature of physical and social hostel settings and hostel life to HIV infection risk. We sought from the data the question of how physical insecurity and the vulnerability of female residents would increase their HIV infection risk.

## Results and discussion

To answer the research questions we posed, we present findings on hostel HIV infection risk factors, on beliefs, values, traditions and sexual relations amongst hostel residents that may underlie the risk factors and on what tertiary institutions could do to mitigate the impact of these risk factors. We do this by presenting the integrated questionnaire and focus group interview data according to 12 thematic categories of tertiary institutional hostel-related HIV/AIDS infection risk factors. These are thematic risk factor categories of policies, rules and regulations on the management of hostels, overcrowding and ‘squatting’ in the hostels, access and security, safety, meeting basic needs, sexual relations, sexual abuse, psychosocial support, HIV/AIDS awareness activities, alcohol and drug abuse, beliefs, attitudes and cultural values and measures that could be taken to mitigate the risk factors.

The results and their discussion are presented according to the format that follows.

### Policies, rules and regulations for managing hostels

Procedures in the form of policies, rules and regulations are pivotal in guiding individuals' behaviour and practice. Findings of this study revealed that the majority of the institutions that participated in this study did not have HIV policies of their own. Although the UNAM developed and introduced its own HIV/AIDS Policy in 2001 and published its Sexual Harassment Policy and Procedures in 2008, there are no rules and procedures restricting hostel residents (whether male or female) from visiting each other any time of the day or night. According to our data, 77% of the respondents agreed that female students may be visited by male students any time of the day or night. In addition, 80% of the respondents agreed that male students can be visited by female students any time of the day or night. When hostel residents were asked about whether there were HIV/AIDS Hostel Policies in their individual institutions, they responded as depicted in Table 3.

**Table 3.** Were there HIV/AIDS policies for hostels at the institutions?

Response	Frequency	Percentage
Yes	79	12.5
No	522	83.0
Not sure	28	4.5
Total	629	100

Respondents who reported the existence of an HIV/AIDS policy were from the UNAM where such a policy has been in existence since 2001. Notwithstanding its existence and its commitment to creating conditions in which HIV/AIDS services, support and care would be provided to students and staff, there was no HIV/AIDS policy for the hostel at the institution.

This implies that HIV/AIDS policies need to be designed for the hostels at all tertiary education institutions.

### Overcrowding and ‘squatting’ in the hostels

Officially, the number of occupants in Namibian tertiary institutional hostels should range from two persons per room for the UNAM and Polytechnic of Namibia to four persons per room for all the then Colleges of Education. However, our findings showed that the number of occupants per room was in a number of cases higher than this. For example, 36.3% of the respondents indicated that the number of hostel occupants per room ranged from four to nine persons per room. Furthermore, because of lack of accommodation, 40% of the respondents indicated that unmarried male and female students ‘squatted’ in the same rooms. In our understanding, this could create risks for HIV infection.

These findings are supported by findings from focus group discussions. These revealed that lack of accommodation forced many students to squat with boyfriends and girlfriends and that some girls were made to co-habit with partners older than themselves. In situations such as these younger girls found it very difficult to make decisions on matters related to sexual intercourse. Furthermore, it was reported that several girls were lured into sexual relationships without commitment in exchange for money to pay for tuition and accommodation off campus. These findings show that many hostel residents became vulnerable to HIV infection because of a shortage of hostel accommodation, poverty and lack of social-economic empowerment. This vulnerability tended to be gender related, with girls being more at risk of HIV infection than boys. This implies that any interventions involving life skills education focusing on abilities such as assertiveness, decision-making and sexual negotiation skills should be sensitive to the situation of female hostel residents (Yotebeing, Halpern, Mitchell & Adimora 2009).

In summary, we wish to highlight the following issues:

1. Scarcity of hostel accommodation in tertiary institutional hostels resulted in ‘squatting’. This posed HIV infection risks when those squatting may be required to pay ‘in kind’ for the accommodation.
2. Focus group discussions revealed that older men or women often flirted with young girls or boys in exchange for accommodation. This practice operated as a ‘sugar daddy or sugar mummy syndrome’. This implied that scarcity of accommodation made many female hostel residents vulnerable to sexual exploitation.
3. Intervention to reduce HIV infection risk behaviours should include empowerment through life skills education for hostel students and an appreciation of how poverty and lack of socio-economic empowerment for girls in Namibia in particular and in Sub-Saharan Africa in general create environments in which HIV infection risks thrive (Maltin & Spence 2000).

In Namibia, findings similar to these have been reported in studies by Palander (2003), Zimba (2003), and Iipinge, Hofnie and Friedman (2004). They have also been reported at Universities in Ghana (Anarfi 2000), South Africa (Barnes 2000) and Zambia (Kelly 2001). Recently, Grannis (2011) has used data gathered from the Sub-Saharan African countries of Botswana, Burundi, Rwanda, South Africa, Kenya, Uganda, Mozambique, Malawi, Tanzania, the Democratic Republic of Congo and Zambia to describe how men's cultural dominance and women's passivity and compliance conspire to put young girls and women at high risk of HIV infection. According to her the main vehicle for HIV infection in this context is transactional sex between girls and women on the one hand and male sexual predators on the other.

### Access to and security in the hostels

In terms of physical access to most of the hostels at the tertiary institutions, 70% of the respondents reported that non-hostel residents can gain unauthorized entry into the hostels at any time of the day or night (see Table 4). In fact, 62% of them reported that non-hostel residents do gain unauthorized entry into the hostels. To exacerbate the situation, 56% of the participants reported that there were no measures in place to prevent unauthorized access to the hostels. In addition, 66% of them reported that campus security guards did not prevent unauthorized access to the hostels.

**Table 4.** Non-hostel residents can easily enter hostel premises at any time of day/night.

Response	Frequency	Percentage
Strongly agree	295	47.5
Agree	136	22
Strongly disagree	104	16.7
Disagree	86	13.8
Total	621	100

Focus group discussions that took place at all institutions in the sample revealed several security lapses. These included the findings that because a number of the hostels were not fenced, they allowed free physical access to anyone at any time of the day or night, there was no control of movement in and out of the hostels, doors to hostel rooms were either broken or could not lock, shower rooms and toilets that were broken and in some cases stained with blood did not have doors in most cases, the security gates on many campuses were open 24 hours and there were no hostel staff to ensure security in most hostels. As a consequence of all this, the majority of the participants were afraid of being infected with HIV in the hostels. In particular, several female participants were afraid of being raped by both strangers and some of their male colleagues in the hostels owing to security lapses.

Moreover, there were no restrictions about male and female students visiting one another's rooms at any time of day and night. Visitors and unauthorized intruders could also enter and leave the hostels as they pleased. Furthermore, because of lack of accommodation, some male and female students ‘squatted’ in the same rooms. This meant that male and female students shared hostel rooms in a number of cases on almost all sampled campuses. When this happened, male and female students were reported using the same toilets and shower rooms.

At one UNAM campus, male and female students were sharing the same hostel block in which shower rooms and toilets were not labelled according to gender. In addition, shower rooms had no doors. This meant that there was no privacy when one was taking a shower. Men and women could also use the same toilets. The implication was that the ablution facilities were co-educational. From these findings, it is reasonable to conclude that insecurity in the hostels created conditions that in all likelihood promoted HIV infection amongst hostel dwellers.

### Safety in the hostels

The majority of participants reported that they were not and did not feel safe in the hostels. About 70% of them felt this way because bathroom doors could not lock and they were afraid of being attacked while taking showers. Furthermore, over 60% of them were afraid of being sexually attacked in their hostel rooms (see Table 5). Over 52% of the participants felt that their fear was heightened by the realization that none of their peers would come to their assistance in the event that they were attacked. To collaborate this, over 52% of them reported that female hostel residents were assaulted by their boyfriends in the hostels.

**Table 5.** Students were afraid of being sexually attacked in hostel rooms.

Response	Frequency	Percentage
Strongly agree	285	46.2
Agree	104	16.9
Disagree	108	17.5
Strongly disagree	120	19.4
Total	617	100

During focus group discussions, participants from all sampled institutions explained their fear of being infected with HIV in the hostels by indicating that they did not want to die as AIDS had no cure. In addition, they did not want to disappoint their parents, lose friends, be stigmatized, destroy their future and not be able to attain their career aspirations. Moreover, they did not like the prospect of being constantly anxious about the future, worried and depressed about becoming a burden to family members and about not being able to have families with children. Furthermore, the prospect of not being able to make tangible contributions to the development of their country bothered several of them.

The lack of safety in the hostels that is depicted in the above findings does not fully capture the fear and hopelessness that we observed in the eyes, gestures, facial expressions and voices of the young people we interacted with during focus group discussions. They appeared helpless as they seemed not to expect their living conditions to change in the near future.

### Meeting basic needs in the hostels

One of the main risk factors that could contribute to HIV infection amongst hostel residents is the desire to meet basic needs for food, clothing, accommodation, tuition and transport to and from campuses. Focus group discussions revealed that several hostel residents (especially female ones) engaged in sexual relationships with older men and women simply to meet their basic needs. In addition, it was revealed that there existed peer pressure at the tertiary institutions that forced several hostel residents to engage in sexual relationships with taxi drivers, ‘sugar daddies’ and ‘sugar mummies’ for monetary and material gain (see Table 6). These findings are consistent with those reported by Grannis (2011) and Kelly (2001).

**Table 6.** Did sexual activities for monetary/material gain occur in the hostels?

Response	Frequency	Percentage
Yes	341	54.2
No	276	43.9
Not sure	12	1.9
Total	629	100.0

### Sexual relations in the hostels

Although over 80% of the respondents agreed that one could have a good relationship with someone from the opposite sex without sexual intercourse, more than 51% of them reported that men and women should experience sex before marriage (see Table 7) and about 49% of them indicated that there was nothing wrong with doing this. Consistent with this reasoning, 66% of the participants reported that some students lived with partners in their hostel rooms without permission from authorities managing the hostels (see Table 8). About 52% of them reported that such partners were in a number of cases not students.

**Table 7.** Men and women should have sexual experience before marriage.

Response	Frequency	Percentage
Strongly agree	171	27.6
Agree	148	23.9
Disagree	102	16.5
Strongly disagree	199	32
Total	620	100

**Table 8.** Some residents lived with partners in hostel rooms without permission.

Response	Frequency	Percentage
True	406	65.5
False	214	34.5
Total	620	100

To elicit more information on how sexual relations in the hostels could promote HIV infection, we, in focus group discussions, asked two questions. First, we asked about what students did in the hostel that could promote HIV infection. Second, we asked how hostel life could promote HIV infection.

In response to the first question, participants from all sampled institutions reported that several students tended to have multiple partners from within and outside the colleges with whom they had concurrent relationships. The other activity was the tendency for male students to go out, drink alcohol and pick up girls and bring them to college campuses and have sex with them without protection. Female students also were observed to have sexual relationships with sugar daddies and others under the influence of alcohol.

Moreover, students from the institutions reported that there was a lot of peer pressure to engage in unprotected sex. This was in the form of bringing partners into rooms and encouraging others to do the same, introducing friends to sugar daddies, communicating the message that exchanging sex for money to buy food, clothes and cosmetics was all right, sleeping around was all right, going out with sugar daddies who did not use condoms was in order, having a boyfriend or a girlfriend was the ‘in’ thing, being picked up in posh cars by older persons boosts one's reputation and that popularity depended on having several partners at the same time. Furthermore, it was reported that sharing shavers and hair cutters, practices that were engaged in by male students, could promote HIV infection. Some female hostel residents' tendency of using pills instead of condoms to prevent pregnancy may in fact promote HIV infection.

The message that comes from these findings is that a culture of reckless promiscuity in which sex is used as a source of material goods, good reputation, popularity, significance and worth poses a major risk for HIV infection in the hostels.

What surprised us was the consistency of these findings with those that were reported more than a decade ago. Kelly (2001) synthesized data on seven African Universities' response to the HIV/AIDS pandemic. On the Universities' social life he had this to say:

The case studies …  … show that the culture of campus life appears to be ambivalent about – or even open to ‘sugar daddy’ practices, sexual experimentation, prostitution on campus, unprotected casual sex, gender violence, multiple partners and similar high-risk activities. In the context of HIV/AIDS, student communities with such a culture are in danger of encouraging risk more than safety, thereby abetting death more than life. As a result, a residential university must be regarded as a high-risk environment for the transmission of HIV. (Kelly 2001:13)

This message is reiterated when one considers how life in the hostels could promote HIV infection. For instance, participants from all sampled institutions in our study reported that students in the hostels misuse the unrestrained freedom they have by abusing alcohol and engaging in irresponsible sexual activity. For instance, many residents were observed to freely visit one another's rooms at any time of the day or night. To buy good food, clothes that are in fashion and to keep up with the lifestyles of students who come from rich families, some female students engaged in prostitution and fell prey to sugar daddies who prowled around hostel campuses looking for young girls to flirt with. Moreover, some senior male students in the hostels took advantage of first-year female students whom they forced into relationships.

### Sexual abuse in the hostels

Only 28.2% of the respondents answered in the affirmative when asked about whether sexual harassment was common in the hostels. Respondents in focus group discussions indicated that the harassment that took place was perpetrated by non-hostel dwellers who normally acted under the influence of alcohol. From Table 9, it can be deduced that several ‘sugar daddies’ engaged in the harassment.

**Table 9.** Did older men have sexual relationships with younger female hostel residents?

Response	Frequency	Percentage
Yes	454	72.2
No	166	26.4
Not sure	9	1.4
Total	629	100

Another aspect that could be regarded as sexual harassment and sexual abuse was reported in the focus group discussions. This involved senior students coaxing, taking advantage of and forcing young girls (especially first years) to have sexual intercourse with them. These activities were facilitated by the fact that entry into girls' dormitories without permission by senior male students (who were frequently under the influence of alcohol) was reportedly very common. In our judgement, these activities would promote HIV infection.

### Psychosocial support

We sought to find out whether systems were in place in the hostels to provide psychosocial support that was aimed at either preventing HIV infection or mitigating repercussions of behaviours that may promote the infection. The questionnaire data revealed that about 51% of the respondents reported the non-existence of HIV/AIDS committees organized to provide counselling services and information to prevent the spread of the pandemic. Whereas 59% of the participants reported that new hostel residents were not oriented on the dangers of HIV infection in the hostels (see Table 10), 72% of them indicated that victims of sexual harassment received no support from hostel management (see Table 11) and 75% of them reported that no support mechanisms were in place to counsel residents who became victims of sexual abuse.

**Table 10.** New residents were oriented on dangers of HIV infection in the hostels.

Response	Frequency	Percentage
Strongly disagree	238	38.8
Disagree	125	20.4
Agree	135	22.0
Strongly agree	116	18.8
Total	614	100

**Table 11.** Hostel residents who became victims of sexual harassment received support from hostel management.

Response	Frequency	Percentage
True	170	28.2
False	432	71.8
Total	602	100

It is clear from these findings that little psychosocial support was provided to hostel residents who might need it for either protecting themselves from HIV infection or for therapeutic purposes. This places them in a precarious and vulnerable position.

### HIV/AIDS awareness activities in the hostels

Findings from focus groups discussions revealed that there were few HIV/AIDS awareness activities taking place in the majority of the institutions visited. In some cases (especially in the then Colleges of Education) no HIV and AIDS awareness clubs were in existence (see Table 12). Where HIV and AIDS awareness clubs existed, activities were participated in by few students and therefore not effective.

**Table 12.** Did HIV/AIDS prevention and counselling activities take place in the hostels?

Response	Frequency	Percentage
Yes	184	29.3
No	429	68.2
Not sure	16	2.5
Total	629	100

Although the UNAM has made significant progress in terms of involving students in its HIV/AIDS awareness programmes through the ZAMANAWE (an acronym that stands for Zambia, Malawi, Namibia and Western Cape), peer counsellors and peer educators, it seems many of their awareness programmes placed more emphasis on basic knowledge of HIV and AIDS such as abstinence, condom use and sticking to one partner and less emphasis on behavioural change or life-skills education.

### Beliefs, attitudes and practices

To answer our second research question, we enquired, during focus group discussions, about beliefs that would promote HIV infection amongst hostel residents. To capture the essence of what came out of the discussions, we present beliefs, practices and dispositions that could promote HIV infection in thematic form and textual form as follows:

The beliefs and ways of reasoning presented in Table 13 were expressed in varying degrees by hostel residents from all sampled tertiary education institutions. As indicated earlier in this article, this catalogue of thoughts and beliefs depicts and justifies a promiscuous sexual culture that would certainly promote HIV infection and the spread of AIDS. This does not mean that there were no hostel residents who did not practice sexual prudence. There were, however, ways of reasoning and behaviour that were overshadowed by a pervasive peer culture of promiscuity and women's vulnerability to HIV infection. Moreover, the message that these beliefs and dispositions communicate is the urgent need to engage in serious HIV/AIDS prevention activities amongst hostel residents.

**Table 13.** Examples of HIV infection risks that were expressed in focus group discussions.

Theme of HIV infection risk	Examples of HIV infection risks under each theme
Fatalistic thinking	HIV/AIDS came for human beings – be brave
Myths about HIV infection	Doing sex one time without a condom will not cause HIV infection; Having sex with a virgin makes one HIV negative
False assumptions	Love without sex is not love; Your thing will be rotten without sex; If you do not have sex early in life, you will not do sex well later in life; A circumcised man is protected from HIV infection and he cannot infect anyone with HIV; Some girls believe that to keep a boyfriend they should get pregnant; If you do not get involved in sex while young, you will go mad. You will be mentally healthy if you have sex; It is not enjoyable to have one partner. Enjoy different tastes from different tribes and countries
Wrong information on HIV infection	Trust and faithfulness are not workable. You are faithful. Do sex. Infected! Sex is a basic need. Doing it fulfils a need whether safe or not; HIV does not exist. It is not real; Boys believe that a healthy looking person is HIV negative; Boys believe that fat girls are HIV negative
Peer pressure	To avoid shame around friends, men should have sex; You are not a real man if you do not have a girlfriend. You are useless; If you do not have sex, you are a coward
Gender vulnerability	Boys only to have a say in sexual matters; Because women are under men, they should obey when men want to have sex without condoms; Women think that they should obey men. How else can you understand female students sleeping in men's hostels; Some girls believe that if a man uses a condom, he does not care for you; Some girls believe that to prepare for disappointments, have more than one partner. If one disappoints you, go to the next one
Practices	Have sex without a condom because with it you feel nothing; Young people should experience sex before marriage; If you are afraid of losing your partner, have casual sex with him or her to save the relationship; You should prove how good a girl is in bed; Culturally, it is not a problem to have more than one partner

At the conceptual level, as depicted in Table 13, this manner of reasoning communicates stereotypical and fatalistic thinking, myths about HIV infection, false assumptions, a belief and value system that would promote HIV infection, wrong information about HIV infection and the basis of peer pressure to engage in unprotected sex.

To provide additional examples based on risks that are not given in Table 13, stereotypical and fatalistic thinking was displayed when participants made statements such as

practice makes perfect; using a condom is a sin; sex is nature – you add a condom, you interfere with nature; real men should sleep with many girls; enjoy sex while you are alive, even if you protect yourself, you will die anyway; you become famous when you have more than one girl friend.

Whereas myths about HIV/AIDS were expressed when participants stated: ‘have sex to get rid of pimples; young boys and girls do not have HIV’, false assumptions were exemplified by the statement: ‘You are not beautiful if you do not have sex.’ A belief system that could promote HIV infection was illustrated when participants indicated:

it is all right to have sex with your boyfriend without a condom; you will kill babies if you use condoms; sex is part of love – to show that you love him or her, do it; some tribes prohibit the use of condoms; if you do not have a girlfriend, you are gay.

### Alcohol and drug abuse in the hostels

Alcohol abuse was recognized as a wide-spread social problem in all the tertiary institutions that participated in this study. It is commonly known that alcohol abuse is linked to violence, abuse and various types of risk-taking behaviours, including sexual risk-taking behaviours (UNICEF 2006).

The findings of the focus group discussions revealed that there was little control of alcohol consumption on the hostel premises. This resulted in a situation where a number of students residing in the hostels abused alcohol and commonly engaged in promiscuous sexual activities under the influence of the substance. In addition, it was reported that older men used alcohol as a bait to lure younger girls to engage in unprotected sex. Under the influence of alcohol such female students lost control and ended up sleeping with strangers. In addressing a situation such as this, the *Psychosocial AIDS risk reduction model* posits that behavioural change occurs when ‘risk-potential producing situations’ are altered (UNAIDS 1999:6). For this to happen in the hostels, mechanisms should be put in place to address the rampant abuse of alcohol.

### Measures to be taken in order to mitigate the risk factors

We asked participants about what they thought could be done to mitigate the HIV risk factors that were inherent in the hostels. We highlight a number of suggestions that emanated from Focus Group discussions that we undertook at all sampled institutions in the manner that follows. We do this with the understanding that these suggestions should be considered as part of the main recommendations of the study.

#### Provision of HIV/AIDS prevention, counselling and psychosocial support services in the hostels

The suggestion that adequate HIV/AIDS prevention, counselling and psychosocial services should be provided in the hostels was expressed frequently in focus group discussions. It was felt that there was a need to further educate the youth in the hostels about HIV infection risks in regular seminars, workshops, audio-video media, posters, sermons, orientation programmes at the beginning of each year and HIV/AIDS clubs. In these activities, hostel residents should be trained in condom use, attitude and behaviour change, abstaining from sexual activities, appropriate sexual relations, morals and Christian values. In addition, it was urged that social workers, HIV-positive individuals, lecturers and pastors should provide psychosocial counselling on assertiveness, self-concept enhancement, sexual relationships guidance, handling peer pressure, voluntary testing and on Christian lifestyles. For these activities to take place, tertiary institution mangers should create structures, where they do not exist, solely dedicated to the welfare of student residents.

#### Provision of security in the hostels

It was pointed out that adequate security measures should be put in place in all hostels. These measures should include enforceable control of access to hostels through the use of rules and regulations, electronic access cards, a properly managed matron system, an effective security guide regime, a functioning system of visiting hours that would regulate the locking of hostel premises and a hostel access monitoring system.

#### Provision of non-toxic entertainment opportunities on campuses

Social clubs (e.g. drama clubs), sports activities and educational movies should be provided to hostel residents – particularly during weekends.

#### Banning of the sale of alcohol on campuses

The majority of participants strongly advised that to prevent antisocial sexual behaviour engaged in under the influence of alcohol, the selling of alcohol on all educational institutional campuses should be banned. In addition, the consumption of alcohol on campuses should be controlled. To strengthen this, alcohol rehabilitation services should be made available to students who need them.

#### Engaging all stakeholders in the provision of basic needs and adequate hostel accommodation to needy and vulnerable hostel residents

Participants suggested that managers of the sampled education institutions, parents/guardians, civil society, captains of industry, educational labour unions, scholarship donors, communities and student leaders should be sensitized on HIV infection risks in the hostels that implicate particularly needy and vulnerable hostel residents. Based on such sensitization, the stakeholders should work out mechanisms of meeting basic needs of vulnerable students in the hostels and seriously explore ways of increasing the provision of safe hostel accommodation for students. Practically, this implies that the public sector, private sector, civil society, the donor community and other development partners should be engaged to mobilize material and financial resources needed for increasing accommodation capacity for tertiary education students. Parents/guardians, in particular, should be actively involved in the lives and social welfare of their children residing in the hostels.

#### Safety assurance in the hostels

A number of participants urged educational institutional managers to ensure that special protection was granted to female hostel residents who were frequently harassed and taken advantage of by some of their male colleagues, sugar daddies and hostel intruders. This could be done by ensuring that access to hostels for female students is strictly controlled and that such hostels are adequately managed by a functioning matron system. This means that access to hostels for female students should be strictly monitored as is the case in many Universities in the Southern African Development Community region and elsewhere.

## Recommendations

In our view, the suggestions pertaining to access to and security in the hostels, safety in the hostels and psychosocial support that participants made in the preceding section of this report capture a number of important recommendations on which the managements of the UNAM (including the former Colleges of Education that have merged with it) and the Polytechnic of Namibia ought to take urgent action. In addition to these suggestions, we make the recommendations that follow according to the thematic risk-factor categories we have used in the report. Furthermore, we make some recommendations on youth development that we can deduce from the findings of this study.

#### Recommendations based on sexual relations exhibited by hostel residents

1. To discourage the operation of a culture of reckless sexual promiscuity that promotes HIV infection, educational institutional hostels should be designed, organized and run in such a way that vulnerability to sexual exploitation is reduced and residents are sensitized to practice restraint in sexual matters. This means that hostels should become controlled living environments in which residents know their boundaries. By making this recommendation, we are not naïve about the fact that hostel residents can be infected with the HIV virus outside the boundaries of tertiary education institutions. However, efforts should be made to ensure that physical, psychological and social environments of hostels do not promote HIV infection.
2. The findings of this study support the position that hostels become communities of young people with psychosocial developmental risks and opportunities. The peer culture, according to this study, that seems to operate in these communities supports the development of thought and belief patterns inimical to safe sexual behaviours. We recommend that tertiary institutions should take the functioning of this culture into account when managing hostels.
3. As has been shown in this study, hostels do not exist in a vacuum. They are part of communities in which they are located. We recommend that through consultative workshops and seminars, all stakeholders should be made aware of sexual relational HIV risks inherent in hostel life. Community members in particular should be made aware of their role in combating the risk by discouraging practices such as those of sexual predators in general and sugar daddies in particular.

#### A recommendation pertaining to beliefs, attitudes and cultural values that promote HIV infection in the hostels

1. We recommend that student leaders, with the support of tertiary institutional structures mandated to ensure the wellbeing of students, should employ peer group strategies to ameliorate adverse effects of stereotypical and fatalistic thinking, myths about HIV infection, false assumptions, and a belief and value system, all of which promote HIV infection.

#### Recommendations on youth development issues in the fight against HIV infection

1. The findings of this study enable us to argue against the assertion that hostel residents are adult enough to make wise decisions when engaging in sexual relations and when relating to each other in an understanding and humane manner. From the findings of this and other studies in the literature on youth development, young adults are explorers and experimenters who are susceptible to misinformation, myths and half-truths – a dangerous combination when it comes to HIV infection. Given this understanding, we recommend that development-related evidence-based strategies be used when organizing living spaces for young people in the hostels. How young people think and behave should be the basis of such strategies.
2. From a Vygoskian perspective, the findings of this study point to the need to provide hostel residents with some social-economic and psychosocial support structures to enable them to fend off HIV infection risks. They need this kind of support from their parents or guardians, lecturers, fellow students and institutional managers. For instance, to avoid taking risks in the process of meeting basic needs of shelter, clothing and food, hostel residents require social-economic scaffolding. In a similar way, they require psychosocial support structures when they are sexually harassed and forced to participate in sexual orgies. Based on this reasoning, we recommend that stakeholders such as parents, guardians, lecturers, Student Representative Council members and managers of institutions should provide those support structures.

### Conclusion

The main message of this article that we wish to communicate is the presence of severe cognitive, attitudinal, cultural and behavioural HIV infection risk factors that were uniquely expressed in the hostel residence context. These were in the form of overcrowding and lack of security, safety, psychosocial support and basic needs. Based on the discussion of the results we have, from a youth developmental perspective, offered ways of mitigating the effects of the risks. We urge the managers of the tertiary education institutions to seriously consider and implement the findings and recommendations of the study on which this article is based. Moreover, we suggest that further research in the area be conducted to extend our understanding of sociological, cultural and psychosocial ramifications of life in University student residences that may promote HIV infection and the spread of AIDS.
